# Effect of Residual Stress on Thermal Deformation Behavior

**DOI:** 10.3390/ma12244141

**Published:** 2019-12-10

**Authors:** Tomohiro Sasaki, Sanichiro Yoshida, Tadashi Ogawa, Jun Shitaka, Conor McGibboney

**Affiliations:** 1 Graduate School of Science and Technology, Niigata University, Niigata 9502181, Japan; f18b086e@mail.cc.niigata-u.ac.jp (T.O.); as.a511agrza@gmail.com (J.S.); 2 Department of Chemistry and Physics, Southeastern Louisiana University, Hammond, LA 70402, USA; syoshida@selu.edu (S.Y.); conor.mcgibboney@selu.edu (C.M.)

**Keywords:** non-destructive testing, optical interferometry, residual stress, thermal expansion, dissimilar bonding

## Abstract

This paper discusses a non-destructive measurement technique of residual stress through optical visualization. The least amount of deformation possible is applied to steel plates by heating the specimens +10 °C from room temperature for initial calibration, and the thermal expansion behavior is visualized with an electronic speckle pattern interferometer sensitive to two dimensional in-plane displacement. Displacement distribution with the thermal deformation and coefficient of thermal expansion are obtained through interferometric fringe analysis. The results suggest the change in the thermal deformation behavior is affected by the external stress initially applied to the steel specimen. Additionally, dissimilar joints of steel and cemented carbide plates are prepared by butt-brazing. The residual stress is estimated based on the stress dependence of thermal expansion coefficient.

## 1. Introduction

In recent years, joining of dissimilar materials has been a subject of interest, in order to provide highly functional structures through a multi-material design. Meanwhile, with the diversification of various methods of joining materials and their shapes, the evaluation of bondability and reliability is becoming increasingly important. In particular, at the joining interface of a dissimilar joint, the difference of thermal expansion induces a high residual stress, leading to degradation of joint strength. Thus, the residual stress measurement induced by the joining process is of a technical nature, as well as a fundamental issue. Several techniques which utilize X-ray [1] or neutron diffraction [2] and acoustoelasticity [3,4] are available for the measurement of the residual stress in a non-destructive way. These methods allow accurate measurement at the microscopic level, while the measurable area is limited to a few millimeters per square at maximum; a more time-consuming process can be applied to a wider area. In addition, the diffractometry, which measures the crystal lattice strain, cannot be applied for the measurement of non-crystalline materials such as polymers, or composite materials. Optical interferometric techniques are widely used for residual stress analyses in conjunction with semi-destructive techniques such as the sectioning, hole drilling, and contour methods. [5,6,7,8,9,10,11,12,13,14,15,16,17,18,19,20]. In these methods, the material around a residually stressed area is removed step by step. At each step, the displacement resulting from the last material removal is measured with the optical interferometry. Some authors use digital holography [7,8,9] and other use ESPI [10,11,12,13,14,15,16,17,18] for the optical technique. Recent studies report non-contact methods to relieve the residual stress using laser ablation [9] or laser annealing [18]. While both techniques commonly evaluate the displacement based on the corresponding change in the optical phase, they have fundamental differences in the principle of phase evaluation. This difference can make either technique suitable or unsuitable for a given application. Often, the advantages and disadvantages of the two techniques compensate each other. It is worthwhile taking some time to discuss the principles of phase evaluation for the two techniques.

Digital holography identifies points on the object via the amplitude and relative phase of the reference laser beam and object beam. Here the relative phase is proportional to the difference in the optical path of the object point from the light source via the reference beam and that of the object beam. Both beams have well-defined wavefronts. Thus, the relative phase can be absolutely evaluated for a given point on the object. By knowing the amplitude and phase for all of the points on the object, it is possible to reconstruct the shape of the object. When the object undergoes deformation, the corresponding displacement of the points on the object can be evaluated through the comparison of the reconstructed shape before and after the deformation. This principle works well if the wavefronts of the reference beam and the object beam are well defined. In some applications where the object surface is not optically flat, so-called speckles are formed on the image plane. Speckles are formed by interference of multiple rays coming from the object surface falling on the same spot on the image plane. The formation of speckles compromises the accuracy of the phase evaluation, as it deforms the object beam’s wavefront [21,22].

ESPI evaluates the relative phase between a pair of speckles formed on the same spot on the object. Here the paired speckles are formed by one of the mutually interfering pair of laser beams and therefore contains the phase value related to the optical path length from the light source. Although the phase of a speckle is not absolutely known, the relative phase difference between the paired speckles changes in proportion to the relative optical path length change associated with the displacement of the object. For instance, consider that the object undergoes displacement such that the point on the object where the paired speckles are formed increases the optical path for one laser beam from $\phi_{1}$ to $\phi_{1}+\Delta \phi_{1}$ in phase, and decreases the optical path for the other laser beam from $\phi_{2}$ to $\phi_{2}-\Delta \phi_{2}$. The phase difference between the two speckles changes from $\phi_{1}-\phi_{2}$ to $\left(\phi_{1}+\Delta \phi_{1}\right)-\left(\phi_{2}-\Delta \phi_{2}\right)=\left(\phi_{1}-\phi_{2}\right)-\left(\Delta \phi_{1}+\Delta \phi_{2}\right)$ (see Section 2.4 for details). Thus, by subtracting the interferogram formed after the displacement from that formed before the displacement electronically, one can form dark fringes where the relative phase difference $\Delta \phi_{1}+\Delta \phi_{2}$ is an integral multiple of 2.

In the present application, we are interested in the change in the elastic modulus due to residual stresses. It is well known that compressive residual stress increases the elastic modulus, and tensile residual stress decreases it. Acoustoelasticity utilizes this fact and detects residual stresses from a change in the acoustic velocity [3]. In our case, ESPI measures the in-plane displacement that object points undergo in response to an external load. Since the stress due to the external load is uniform over the specimen (because the load is static), the resultant strain (differential displacement) is greater than the nominal value if the elastic modulus is lowered by a tensile residual stress [23,24,25]. Conversely, the resultant strain is less if the elastic constant is elevated by compressive residual stress. For the phase evaluation for this analysis, ESPI is more advantageous over digital holography for the following two reasons. First, since we are interested in evaluating the displacement associated with the external loading, it is unnecessary to evaluate the absolute value of the phase before and after the loading separately. Thus, the advantage of digital holography that it can determine the phase absolutely for a single state is not important. Second, the surface of the welded specimen is optically rough. Hence speckles are formed on the image plane of the digital imaging system. This will compromise the phase evaluation by digital holography. On the other hand, since ESPI uses speckles for the phase evaluation, the formation of speckles is not a disadvantage, but rather necessity.

It should be noted that residual stress can vary spatially sharply. Therefore, a high spatial resolution is essential. Digital Image correlation [19,20], although it is handy and fast, involves statistical treatments and that lowers the spatial resolution. It is less advantageous to the present application.

Regardless of the method, the difficulty in nondestructive evaluation of residual stress arises from the fact that the residual stress is locked in the material. Direct measurement of residual stress as a stress essentially involves a process to break the locking mechanism, which in essence means that the process is destructive. The present study focuses on thermal expansion to apply a reversible (elastic) deformation. We evaluated residual stress by locally heating the specimen and analyzing the resultant thermal expansion behavior. The experiments are performed in two steps. In the first step, we examine thermal expansion of the material for an initial calibration, in order to know the influence of the external stress initially applied on the deformation behavior. For the initial calibration, the specimen is gripped by a tensile machine. ESPI sensitive to two-dimensional in-plane displacement is used to visualize the minimal thermal deformation behavior in the temperature range of +10 °C in real time. In the second step, the visualization technique is applied to a dissimilar joint not gripped by a tensile machine. Residual stress, induced by the dissimilar joining, is discussed based on the thermal deformation from the initial calibration.

## 2. Experimental Procedure

### 2.1. Principle of Operation

The principle of operation is as follows. The process of the present residual stress evaluation consists of calibration and evaluation procedures. In the calibration procedure, the specimen is mounted to a tensile machine with various initial tensile loads. The grips of the tensile machine are kept stationary for each tensile load. The initial tensile load mimics residual stress described in Section 2.2, where the stationary grips of the tensile machine correspond to the locking mechanism of the residual stress.

Consider a plate specimen attached to a tensile machine with a certain load being applied, and then spot heat the specimen. The material tries to thermally expand but due to the constraint provided by the tensile machine’s grips, it cannot expand as much as the case when the specimen is free of the constraint. Consequently, the apparent thermal expansion per unit increment of temperature is lower than the case of free thermal expansion (the nominal thermal expansion). The deviation from the nominal thermal expansion is greater when the fixed constraint, due to the tensile machine’s grip, is greater. The greatest deviation is expected when the distance between the tensile machine’s grip is the same as the specimen’s length before the heating. Under this condition, when the material tries to thermally expand, the tensile machine’s grip resists to the greatest extent. When the initial tensile load is greater, on the other hand, the thermal expansion has more room to elongate. Thus, it is expected that as the initial tensile load increases the apparent thermal expansion approaches the nominal value. Our experiment demonstrates this effect as discussed later.

Thus, in the calibration stage, we attach a metal plate specimen to a tensile machine, spot heat the specimen, and measure the resultant strain with the ESPI setup as well as the temperature of the specimen. We repeat this measurement, varying the applied tensile load by adjusting the distance between the two grips of the tensile machine. From the measured strain and temperature, we can evaluate the apparent thermal expansion coefficient. During the measurement of each applied load, the positions of the tensile machine’s grips are unchanged. Therefore, the apparent thermal expansion evaluated from this procedure indicates its dependence on the initial stress applied by the tensile machine. Please note that the spot heating relaxes the initial stress applied by the tensile machine as the material thermally expands. Thus, the stress changes as the temperature rises. The information we try to obtain from the calibration experiment is the apparent thermal expansion coefficient as a function of the initial stress.

In the evaluation stage, we test an actual, dissimilar butt-welded specimen without mounting it to a tensile machine. The spot heating and strain measurement are conducted in the same fashion as the calibration procedure. Along the weld line, the difference in the thermal behaviors of the dissimilar material builds residual stress. When this specimen is spot heated, thermal expansion causes strain. The apparent thermal expansion coefficient is expected to depend on the initial stress (the stress before the spot heating is applied). In this case, the initial stress is the residual stress that the specimen is under before the spot heating. Obtaining the apparent thermal expansion coefficient as a function of the initial stress from the calibration procedure, we can estimate the residual stress of the spot.

### 2.2. Thermal Expansion Test for Initial Calibration with Gripped Specimen

**Figure 1:**
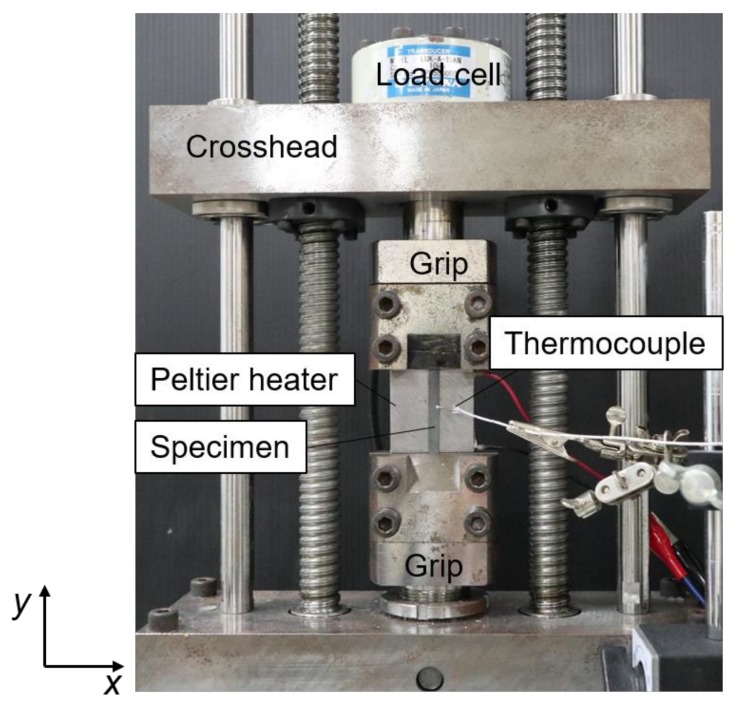
Experimental setup for the thermal expansion test.

The purpose of this test is to explore the relation between residual stress and thermal expansion when the specimen is heated. This information can be used inversely to estimate the residual stress and is described in Section 2.3. A carbon steel plate with a width of 5.0 mm was used for the experiment. The steel plate was annealed in the air at 800 °C for 1 h, in order to release residual stress as received, followed by furnace cooling (the cooling rate is approximately 0.5 °C/s). Specimens with 110.0 mm in length, 5.0 mm in width, and 1.0 mm in thickness, were cut from the annealed plate for tensile tests by electronic discharge machining. A tensile test on the specimen was performed according to ISO 6892 for the tensile characterization. The 0.2% proof stress of the specimen measured in the tensile test was approximately 240 MPa. In the thermal expansion test, external tensile load (initial load) was initially applied to the specimen under displacement control using a cross head of tensile machine (Figure 1). The maximum loading capacity of the tensile machine was 10 kN. The initial load condition was decided based on the corresponding tensile stress range of 10 MPa to 200 MPa, measuring external load with a load cell. Then, the specimen was locally heated from room temperature to about 28 °C at a heating rate of 0.1 °C/s by using a Peltier device attached to the backside of the specimen. The temperature during the experiment was monitored with a thermocouple attached on the surface of specimen. Temperature of the surrounding area including the clamp and the load cell was kept below 22 °C. Deformation behavior during the thermal test was visualized using an ESPI as described in Section 2.4. The thermal expansion test was performed changing the initial load in the step of 10 MPa for the range up to 40 MPa and 20 MPa for the range of 40 MPa to 200 MPa.

### 2.3. Thermal Expansion Test for a Dissimilar Joint

**Figure 2:**
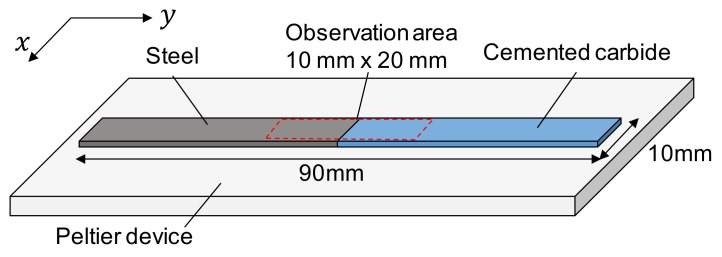
Thermal expansion test for a dissimilar brazed joint.

This test aims to estimate residual stress field near dissimilar bonded interface based on the apparent thermal expansion coefficient under the known applied load condition as described in the previous section. A dissimilar joint of steel and cemented carbide was prepared as a typical example of residual stress caused by external restraint. The difference of thermal shrinkage between the steel and cemented carbide may cause a residual stress at the brazing part. In addition, the cemented carbide/steel joint is used in machine-tool, die and mold industries. In this test, steel and cemented carbide sheets were butt-brazed to each other with binary Ag-Cu filler metal (BAg-24) at the brazing temperature of 750 °C. The joint was cut into a specimen with 90.0 mm in the length, ×10.0 mm in the width ×1.0 mm in the thickness as shown in Figure 2. The dissimilar joint was subjected to the thermal expansion test at a heating rate of 0.1 °C/s. The temperature change applied to the specimen was +10 °C from room temperature.

### 2.4. Electronic Speckle-Pattern Interferometry (ESPI)

**Figure 3:**
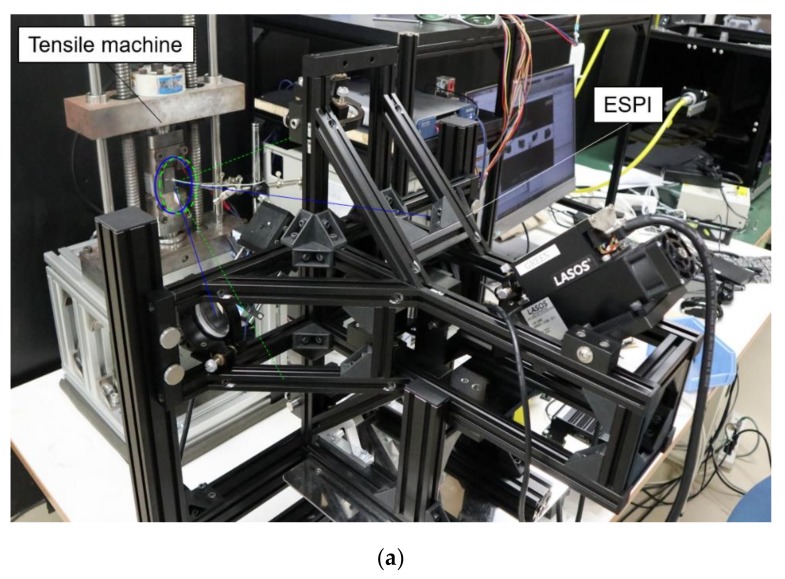

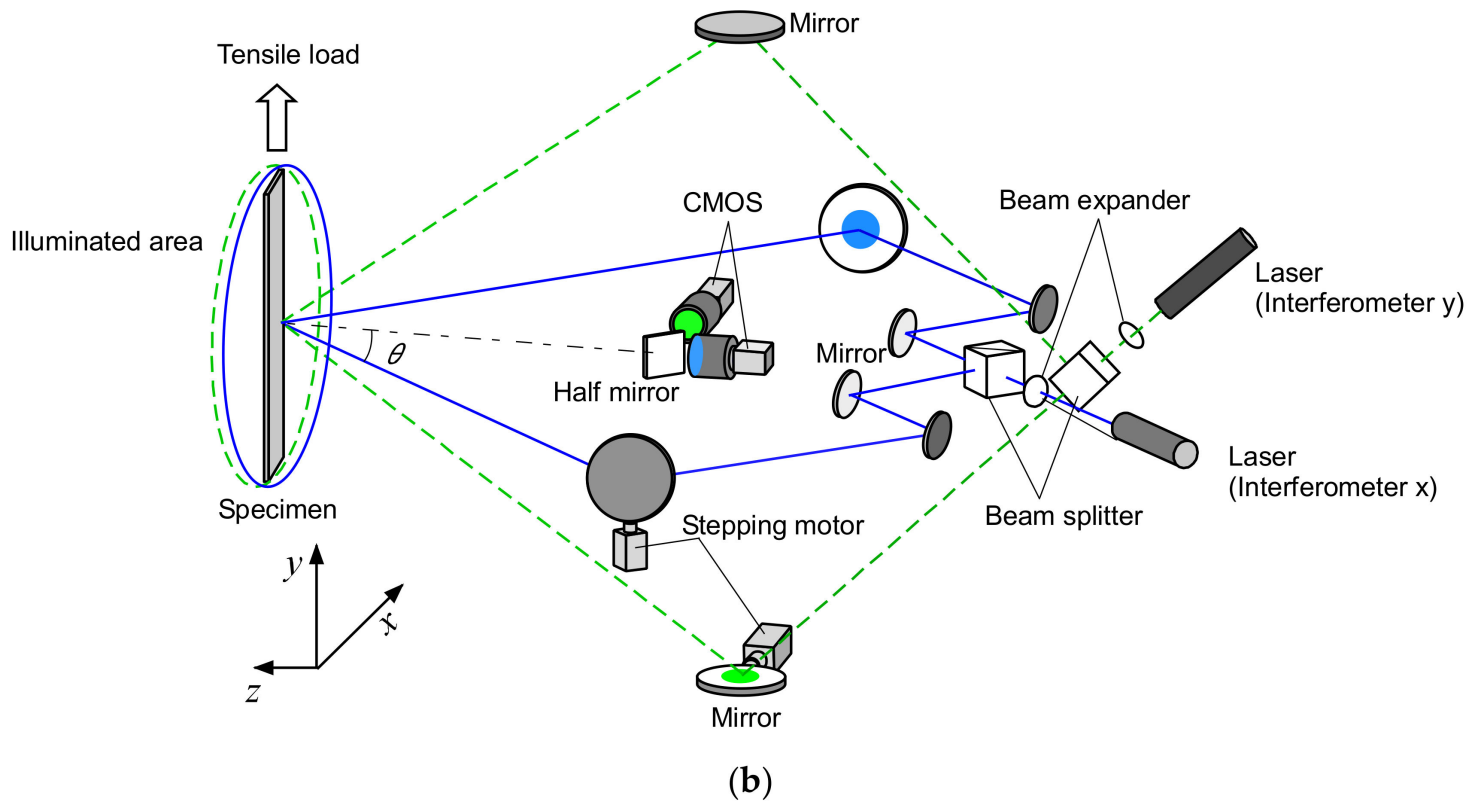
2-D electronic speckle pattern interferometer. (**a**) ESPI apparatus used in this study; (**b**) Optical setup of 2-D electronic speckle pattern interferometer.

Figure 3 shows the setup for visualizing the deformation behavior in the thermal expansion test. The ESPI apparatus was placed in front of the tensile machine as shown Figure 3a. To measure the 2-D displacement field, two optical interferometers were arranged in a parallel direction (*y*-axis) and a perpendicular direction (*x*-axis) to the tensile direction. The optical configurations were sensitive to in-plane displacement component along only *x*-axis and *y*-axis, respectively. Diode-pumped solid-state lasers having different wave lengths of 532.3 nm and 472.9 nm were used for the light sources. Each interferometer consists of a dual beam ESPI [26] as follows (Figure 3b). The laser beam was expanded by a beam expander and split into two paths by a beam splitter and converged to the surface of the specimen via two mirrors in the incident angel, *θ*, of 30.0°. The superimposed speckle pattern generated by the two optical configurations was split into two ways by a half mirror, and captured with two CMOS cameras via optical band path filters. The intensity of the superimposed speckles can be written as follows:

(1) $$I\left(e^{i\phi_{left}}+e^{i\phi_{right}}\right)\left(e^{-i\phi_{left}}+e^{-i\phi_{right}}\right)=2I+2Icos\left(\phi_{left}-\phi_{right}\right)$$

Here, $\phi_{left}$ and $\phi_{right}$ are the phase of speckle due to each interferometric arm. The speckle intensity reflecting from the surface changes depending on the displacement in the sensitive direction due to optical path difference between the two interferometric arms. When the phase of speckle changes by Δϕ/2, its intensity can be written as

(2) $$I\left(e^{i(\phi_{left}+\Delta \phi /2)}+e^{i(\phi_{right}-\Delta \phi /2)}\right)\left(e^{-i(\phi_{left}+\Delta \phi /2)}+e^{-i(\phi_{right}-\Delta \phi /2)}\right)\;=2I+2Icos\left(\phi_{left}-\phi_{right}+\Delta \phi \right)$$

The speckle images are recorded in computer memory and sorted by the direction of configuration, and the intensity difference before and after the deformation are computed by subtracting a frame the deformation from a frame captured at a later frame. The result becomes as follows:

(3) $$2I+2Icos\left(\phi_{left}-\phi_{right}\right)-2I+2Icos\left(\phi_{left}-\phi_{right}+\Delta \phi \right)\;=4Isin\frac{2\left(\phi_{left}-\phi_{right}\right)+\Delta \phi }{2}\sin\frac{\Delta \phi }{2}$$

Since the second term, sinΔϕ/2, becomes zero when Δϕ/2 is equal to an integral multiple of π, the displacement fields in the directions, *x* and *y* on the measured surface can be individually obtained as fringe contours. Displacement components, *u*, *v* in the x, *y* directions on the surface can be obtained by

(4) $$u=\frac{n\lambda_{x}}{2\sin\theta },\;v=\frac{n\lambda_{y}}{2\sin\theta }$$

where n is an integer, $\lambda_{x}$ and $\lambda_{y}$ are the wavelengths of the light source for the two optical configurations, and θ is the incident angle. The strain components can be computed by differentiating the displacement as follows.

(5) $$\left(\begin{array}{cc} \epsilon_{xx} & \epsilon_{xy}\\ \epsilon_{xy} & \epsilon_{yy} \end{array}\right)=\left(\begin{array}{cc} \frac{du}{dx} & \frac{1}{2}\left(\frac{du}{dy}+\frac{dv}{dx}\right)\\ \frac{1}{2}\left(\frac{du}{dy}+\frac{dv}{dx}\right) & \frac{dv}{dy} \end{array}\right)$$

In addition, to make the analyses of fringe contours easier, “carrier fringes” [24] were introduced by tilting a mirror for one interferometric arm. The optical distance of the laser beam reflecting the mirror varies depending on its incident angle. Since the laser beam is expanded by the expander, optical distance on the irradiated surface has a gradient. The carrier fringes orthogonal to the sensitive direction can be introduced by rotating the mirror with a stepping motor. The carrier fringes were introduced at a fixed angle before the thermal expansion test, then the change of fringe pattern during the heating was observed. The resultant fringe contours represent the superimposing displacement obtained from the carrier and the actual displacement due to the thermal deformation. The displacement due to the thermal deformation can be obtained by subtracting the carrier from the measured fringe after the deformation.

## 3. Results and Discussion

### 3.1. Effect of External Restraint on Thermal Deformation Behavior

**Figure 4:**
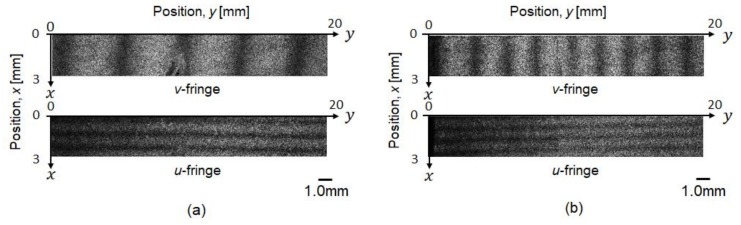
Typical speckle fringes of (**a**) before heating (at 22.0 °C), and (**b**) after heating at the temperature of 24.5 °C under no initial stress condition.

**Figure 5:**
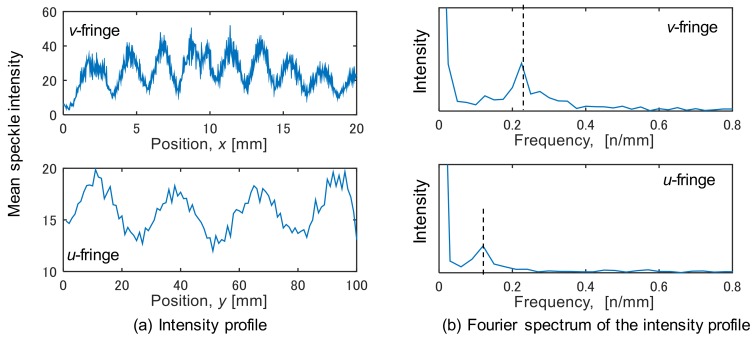
Intensity profile of speckle fringe pattern.

Typical examples of fringe patterns obtained in the heating test are shown in Figure 4. The initial load condition is “0 MPa”, and this test was conducted under non-restrained conditions, where one end of the specimen was not gripped by the tensile machine. Some carrier fringes previously introduced can be seen in the image before the test start (Figure 4a, before heating). The images of “*u*-fringe” and “*v*-fringe” are the respective resultant fringes obtained from the interferometers sensitive to the directions of *x* and *y*. The number of fringes in the observation area increases during heating due to thermal expansion (Figure 4b, after heating). The appearance of fringes implies that displacement difference occurs in the observation area (3 mm × 20 mm). In addition, the fringe is vertical to each sensitive direction, indicating that the deformation was almost uniform in this experiment. To obtain averaged strain in the observation area, speckle intensities were averaged in the direction vertical to the sensitive direction. Figure 5a shows the averaged intensity profile for each direction. The frequency spectrum was then calculated through Fourier transform for the intensity profile (Figure 5b). The peak frequency in the spectrum is indicative of the number of fringes per unit length. The displacement difference corresponding to the number of fringe, i.e., the averaged strain, *ε_yy_*, in the observation area can be obtained by Equation (3). Similarly, the strain *ε_xx_* was obtained from the speckle fringes for the *x* direction.

**Figure 6:**
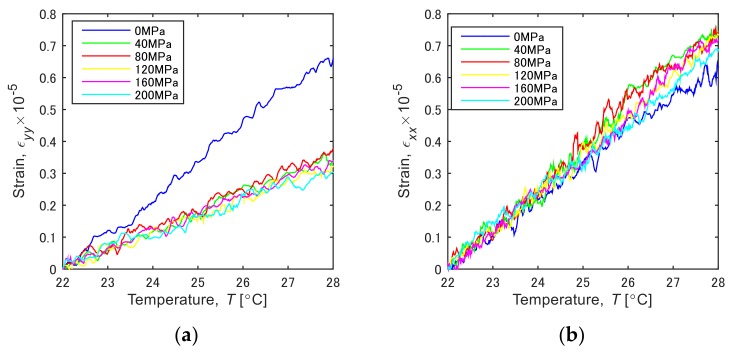
Changes of thermal strain in the directions (**a**) *ε_yy_* and (**b**) *ε_xx_*.

**Figure 7:**
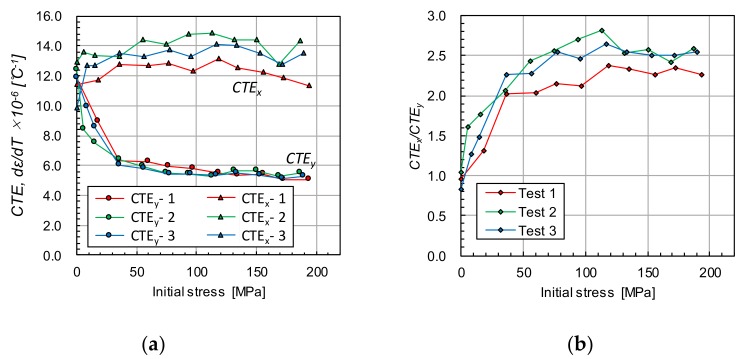
(**a**) Apparent coefficient of thermal expansion, *dϵ/dT*, and (**b**) the direction ratio of the thermal expansion plotted against stress initially applied to the specimen.

Figure 6 shows changes of the mean strain during the heating test in the temperature range of 22–28 °C plotted against the heating temperature. In the condition of initial stress “0 MPa” shown by a blue line. The strain values of *ε_yy_* and *ε_xx_* essentially increase linearly due to the thermal expansion. The value of *ε_yy_* (Figure 6a) under the restrained condition (40–200 MPa), where both ends of the specimen are gripped by the tensile machine, is substantially smaller than the non-restrained condition (0 MPa). In contrast, the value of *ε_xx_* (Figure 6b) is greater than the non-restrained condition. The slopes of the line *dε/dT* indicate the apparent coefficient of thermal expansion in the corresponding direction under the restrained condition (*CTE*). Figure 7a,b plots *CTE_y_* and *CTE_x_*, respectively, to the stress initially applied to the specimen (initial stress). The inclination was calculated by fitting the curve with the least-squares method. The heating test was conducted for three specimens, and each plot indicated in red, green and blue markers shows their results. The *CTE_y_* in the *y* direction decreases depending on the stress initially applied to the specimen (initial stress), and this is less than the coefficient of linear expansion of the carbon steel, 11.7 × 10^−6^ (Figure 7a, *CTE_y_*). In contrast, *CTE_x_* in the *x* direction exhibits an increasing trend under the applied stress condition (Figure 7a, *CTE_x_*). The tensile stress applied in the *y* direction gives rise to compressive stress in the *x* direction due to Poisson’s effect. The slight increase of *CTE_x_* is presumably associated with the compressive stress. Figure 7b plots the direction ratio of thermal expansion, (*CTE_x_*/*CTE_y_*) to the applied stress. The direction ratio is approximately 1.0 under the non-restrained condition (0 MPa), indicating isotropic expansion, then it increases with the increase of applied stress. This fact implies that the thermal expansion behavior changes isotropic to anisotropic depending on the applied stress in the tensile direction. These results show that the principal stress and its direction can be estimated by visualizing the thermal deformation.

**Figure 8:**
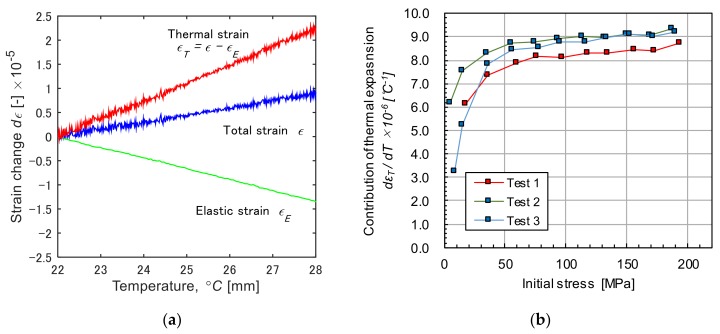
(**a**) Changes of strains during the heating test in the applied stress condition, 100 MPa. (**b**) Contribution of thermal expansion to elastic recovery, *dϵ_T_*/*dT* vs. initial stress plot.

To discuss the above thermal expansion behavior under the restrained condition, we separately evaluated thermal and elastic strain of specimen. Figure 8a shows a typical example of changes in the strains during the heating test (the initial stress: 100 MPa). Three lines indicate the total strain and the elastic strain, and the thermal strain, respectively. The tensile stress was applied to the specimen using the cross-head of a tensile machine. In this situation, the external stress measured by the load cell is balanced with a resistant stress due to the elastic strain of specimen. When heating the specimen, the elastic strain decreases attributed to the increase of thermal strain, i.e., the elastic recovery, occurs with the thermal expansion. Thus, the total strain, *ϵ*, which is directly measured with the ESPI, is considered to be the sum of elastic strain, *ϵ_E_*, and the thermal strain, *ϵ_T_*. Assuming that the elastic modulus, *E*, of the specimen is constant at 207 [GPa] (carbon steel) in the temperature range of 22–28 °C, the *ϵ_E_* can be estimated by *σ/E*, where σ is the external stress calculated from the load measured by the load cell. At the same time, *ϵ_T_* can be obtained by *ϵ* − *ϵ_E_*. In Figure 8a, the elastic strain exhibits a linear decrease during the heating test, while the total strain shows a positive inclination. This indicates that the thermal strain has a greater contribution to the total strain. Figure 8b plots the contribution of thermal expansion to the elastic recovery, *dϵ_T_*/*dT* obtained from the inclination of *ϵ_T_* in the tensile direction to the initial applied stress. The markers indicated in red, green, and blue show the results for the three tests. The value of *dϵ_T_*/*dT* is close to zero at the lower applied stress, showing that the change in *ϵ_E_* is almost equal to that of *ϵ_T_*. Because the thermal expansion is completely restrained or pressed down by the tensile machine, the specimen cannot expand. Then, the *dϵ_T_*/*dT* increases with the increase of initial stress. This indicates that the elastic recovery of the surrounding area occurs under the larger tensile stress, resulting in the appearance of thermal expansion of the specimen. The elasticity of material has been found to be dependent on the applied stress due to the non-linearity between the interatomic force and the interatomic distance [3], in particular, it is known to decrease at a larger tensile strain. Table 1 shows the stress drop values during the thermal expansion tests. The results indicate that the elastic recovery tends to be greater with an increase in the initial stress. One possible explanation for such a greater elastic recovery is that non-linear elasticity appeared in the thermal expansion test (see Appendix A).

### 3.2. Thermal Deformation of Dissimilar Joint

**Figure 9:**
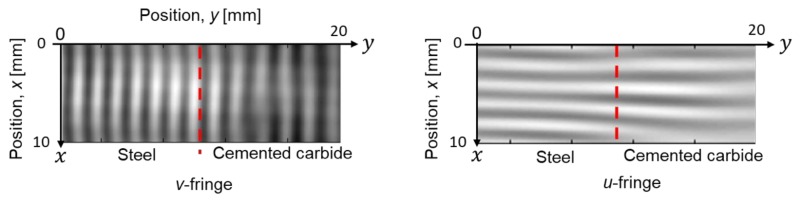
Speckle fringes in thermal expansion test at the heating temperature of 32.0 °C for a dissimilar joint. The temperature change is 10 °C.

**Figure 10:**
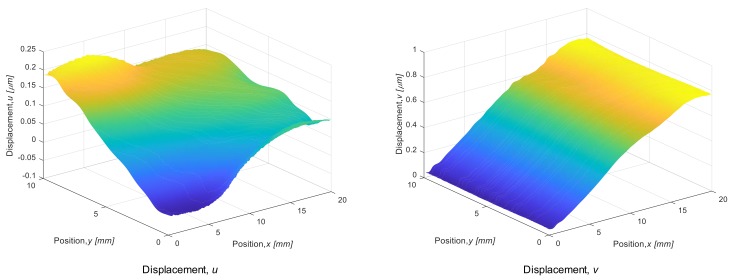
Displacement map obtained from fringe patterns shown in Figure 9.

**Figure 11:**
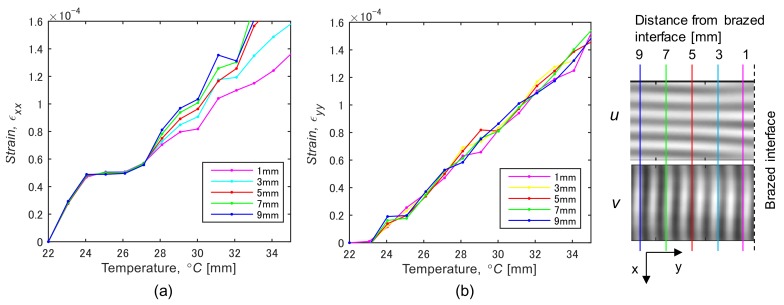
Thermal strain of steel side for the dissimilar joint. (**a**) *ε_yy_* and (**b**) *ε_xx_*.

Figure 9 shows filtered ESPI fringe patterns for the dissimilar joint. The patterns respectively represent the displacement contour in the directions of *y*, and *x* (*v*-fringe and *u*-fringe). The fringe pattern obtained in ESPI includes intense noise at a high frequency, referred as “speckle noise”. To remove this high-frequency noise, a low-pass filter from a two-dimensional Fourier trnasform was applied to the original speckle patterns. In the results in Figure 9, a cut-off frequency was determined based on the number of carrier fringe; the number of fringe higher than 1.0/mm was removed. Looking at the fringe pattern in the *x* direction of the steel side, the fringe interval is slightly larger near the bonding interface, indicating smaller strain. In contrast, the fringe interval at the cemented carbide side becomes smaller. Figure 10 shows displacement maps corresponding to the fringe patterns shown in Figure 9. Strain components *ε_xx_*, *ε_yy_* were respectively computed by differentiating the displacement value as described in Section 2.4. Figure 11 shows the variation of thermal strain *ε_xx_* in the steel side during the heating test. The mean strain was calculated from the displacement map for five points: the distances from the brazing interface are 1.0 to 7.0 mm. A difference in the strain, *ε_xx_* (Figure 11a), appears above 28 °C, and the inclination is smaller in the area closer to the bonding interface. Coefficients of linear thermal expansion of steel and cemented carbide are 11.6 × 10^−6^, and 5.7 × 10^−6^ [°C^−1^], respectively. This difference in thermal expansion may cause a tensile residual stress at the steel side in the cooling process of brazing because the thermal shrinkage is affected by the cemented carbide with the smaller expansion. The difference in the thermal strain is smaller in *ε_yy_* compared to *ε_xx_*. This may be because the cemented carbide does not restrain the thermal deformation in the *y* direction. To estimate the brazed induced residual stress by comparing the thermal expansion behavior with the result in the initial calibration, we obtained the apparent CTE for the dissimilar joint. The strain vs. temperature plot was obtained for each measurement point, and the inclination was obtained by fitting the curve with the least-square method.

**Figure 12:**
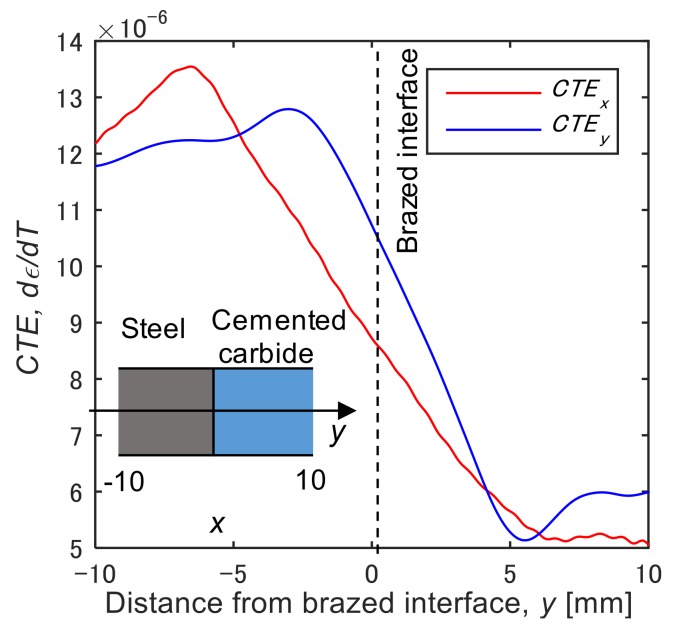
Apparent coefficients of thermal expansion, *dϵ_yy_*/*dT (CTE_y_)* and *dϵ_xx_*/*dT (CTE_x_)* plotted against distance from the brazed interface.

The apparent *CTE_x_* and *CTE_y_* in the dissimilar brazed joint are respectively plotted against position in the *y* direction as shown in Figure 12. The *CTE_x_* has a peak at the position −6.5 mm (6.5 mm away from the brazed interface), then it decreases the bonding interface. A similar trend can be seen in *CTE_y_* in the *y* direction. Both the coefficients are in rough agreement with the coefficient linear thermal expansion of steel at the position far from the bonding interface. The decrease in the apparent CTE near the brazed interface is qualitatively consistent with the result in the thermal expansion test under the restrained condition mentioned above. It can be said that the residual stress acted as the external restraint, resulting in the decrease of CTE. Comparing the range of CTE of the steel near the brazed interface, 8.0 × 10^−6^ to 12.0 × 10^−6^ [°C^−1^] with the apparent CTE shown in Figure 7, the external stress range is approximated to be 0 to 50 MPa, it seems to be smaller than the residual stress generally introduced by the dissimilar brazing. This is presumably due to the fact that the thermal expansion also occurs in the cemented carbide, which restrains the expansion of steel.

## 4. Conclusions

The present study investigated the effect of external restraint on the thermal deformation behavior of steel sheet through visualization using two-dimensional ESPI. Under the restrained condition, the apparent coefficient of thermal expansion decreases depending on the external stress initially applied to the steel. The reason for the negative dependence of thermal expansion is not fully understood at this time. The change of coefficient of thermal expansion is qualitatively observed in the bonding interface of the brazed dissimilar joint. We infer that the residual stress induced in the brazing process affects the thermal deformation behavior. The results indicate the feasibility of residual stress estimation using the visualization of reversible thermal deformation in the temperature range of ±10 °C.

## Figures and Tables

**Table 1:** Stress drop during the thermal expansion test in the temperature range of 22–28 °C.

**Initial stress [MPa]**	10	20	30	40	60	80	100	120	140	160	180	200
**Stress drop [MPa]**	9.1	15.4	35.7	40.6	44.1	44.5	45.9	44.4	47.1	49.6	47.5	50.3

**Figure A1:**
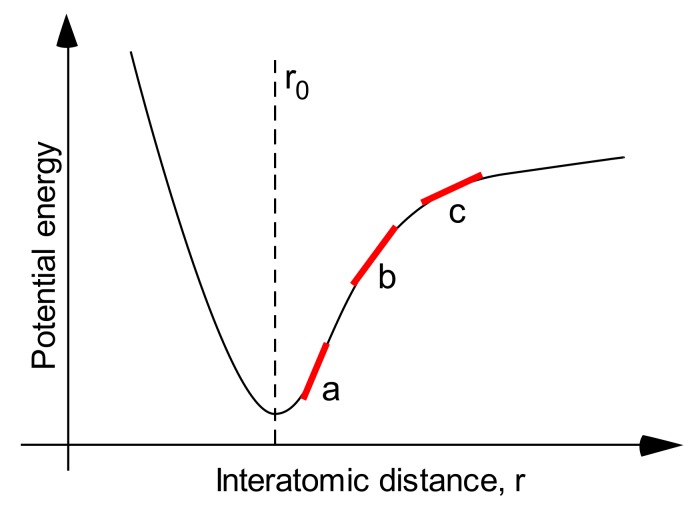
Variation of interatomic potential with interatomic distance, *r.*

## Appendix A

Figure A1 schematically shows the relationship between potential energy and interatomic distance. As the external tensile stress increases, the slope of the potential curve decreases as indicated by the markers a, b, and c. The elasticity of material has been found to decrease at the longer interatomic distance due to this non-linearity of interatomic potential. In addition, when the specimen is spot-heated, the mean interatomic distance increases by thermal expansion.

According to the second law of thermodynamics, the change in the internal energy *dU* satisfies the following equality for elastic deformation (a reversible reaction).

(A1) U=TdS−PdV

Applying this equation to the *x*- and *y*-direction, we can obtain following equations. Here Equation (A1) is perpendicular to the tensile axis and Equation (A2) is parallel. Since the spot-heated part of specimen does negative work, $PdV=-\left|W\right|$ on the non-heated part in the tensile direction.

(A2) $$dU_{x}=TdS_{x}-P_{x}dV_{x}=TdS_{x}$$

(A3) $$dU_{y}=TdS_{y}-P_{y}dV_{y}=TdS_{y}+\left|W\right|$$

Here $P_{x}=0$ because the expansion in the *x*-direction is free expansion. The heating source does not differentiate the directionality, thus we can assume that the internal energy for both directions is the same. This leads to the following relation.

(A4) $$TdS_{x}=TdS_{y}+\left|W\right|$$

As shown in Figure 7a, the expansion in the *x*-direction is always greater than the *y*-direction, thus $TdS_{x}>TdS_{y}$. Furthermore, the difference between them is |*W*|. Furthermore, the difference between them is |*W*|. Figure 7b indicates that the value of |*W*| is greater at the higher initial stress.
